# Dual-Target Bioprocessing Using Oleaginous Microorganisms: Converting Food Waste into Lipids and Biopolymers

**DOI:** 10.17113/ftb.64.01.26.9268

**Published:** 2026-02-15

**Authors:** Zahra Montazer, Kianoush Khosravi-Darani

**Affiliations:** 1 Department of Health and Quality Control, Semnan University, Semnan, Iran; 2 Department of Food Technology Research, Faculty of Nutrition Sciences and Food Technology/National Nutrition and Food Technology Research Institute, Shahid Beheshti University of Medical Sciences, Tehran, Iran

**Keywords:** oleaginous microorganisms, single cell oils, polyhydroxyalkanoates, food waste conversion, integrated bioprocessing

## Abstract

The increasing demand for sustainable alternatives to fossil-derived fuels and plastics has intensified research into microbial platforms that can convert abundant waste resources into valuable products. This review focuses on the emerging field of dual-target bioprocessing using oleaginous microorganisms to produce single-cell oils (SCOs) and polyhydroxyalkanoates (PHAs) from food waste. We discuss key microbial strains, their metabolic pathways, co-production capabilities and substrate preferences. Emphasis is placed on the use of food waste as a low-cost and carbon-rich feedstock, thereby enhancing both economic feasibility and environmental sustainability. We also analyze integrated bioprocess strategies developed to overcome existing challenges, such as yield optimization and metabolic bottlenecks. This dual-production platform addresses the principles of circular economy, facilitating the conversion of waste into high-value bioproducts.

## INTRODUCTION

The increasing global demand for sustainable food resources, non-fossil fuels, and biodegradable packaging materials highlights the need for environmentally friendly and renewable alternatives. Population growth, worsening environmental degradation, and the limited availability of fossil resources are placing growing pressure on current production and consumption systems (*1*). Traditional lipid sources, such as crops and animal fats, require extensive agricultural production, contributing to deforestation, habitat destruction and biodiversity loss. The expected increase in edible oil demand to USD 307 billion by 2029 may require an additional 317 million hectares of cropland by 2050 (*2*). At the same time, depletion of fossil fuels and increasing pollution from plastic waste further exacerbate environmental concerns (*3*, *4*).

Oleaginous microorganisms, including certain yeasts, fungi and bacteria, represent a promising alternative for producing high-value product like single-cell oils (SCOs) and polyhydroxyalkanoates (PHAs) from low-value substrates such as food waste (*5*). This dual-target approach not only addresses the urgent need for sustainable biofuels and biodegradable bioplastics but also aligns with the principles of the circular bioeconomy by enhancing efficiency and reducing environmental impacts (*6*, *7*). Leveraging food waste can significantly decrease production costs, potentially by up to 75 %, improving the commercial viability of microbial oil production (*8*).

This review covers the characterization of microbial strains, metabolic pathways, fermentation strategies, downstream processing, and the techno-economic and environmental advantages of adopting dual-target bioprocessing with oleaginous microorganisms.

## OLEAGINOUS MICROORGANISMS AND THEIR STORAGE METABOLITES

Oleaginous microorganisms are characterized by their ability to synthesize intracellular lipid and polymeric storage materials with a non-polar nature (oleochemicals) from various substrates, including carbon dioxide, sugars and organic acids (*9*). Among the accumulated metabolites, the two most interesting are single-cell oil (SCO) and polyhydroxyalkanoates (PHAs). Both are produced *via* distinct metabolic pathways and are often produced in response to similar environmental triggers, such as nutrient imbalance and carbon excess (*9*, *10*). While most PHA-producing microorganisms are prokaryotes, SCO-producing ones are typically eukaryotes. Notable oleaginous genera with high production include *Cutaneotrichosporon oleaginosus* and *Lipomyces starkeyi*, which have achieved the highest SCO concentrations, reaching approx. 16.77 and 32.7 g/L, respectively, on glucose under nitrogen-limited fed-batch conditions (*10*). Moderate producers such as *Rhodosporidium toruloides* (10–16 g/L) and *Pichia cactophila* (7.1 g/L) show favourable profiles enriched in mono- and polyunsaturated fatty acids, while *Yarrowia lipolytica*, though a lower native producer (2.5–3.1 g/L), offers strong potential for yield improvement through metabolic engineering (*10*), alongside certain bacteria such as *Cupriavidus necator, Bacillus subtilis* and *Pseudomonas* spp. (*11*). These microorganisms can accumulate significant amounts of storage compounds under optimised growth conditions (*12*-*15*).

## DIVERSITY AND BIOCHEMISTRY OF MICROBIAL OILS

A key advantage of oleaginous microbes as lipid-producing platforms is their unique ability to biosynthesize specialized fatty acids (omega-3 and omega-6 groups) that are rare or absent in plants and animals. While fish oil has been the conventional source, concerns about marine resource depletion and quality variability have intensified research into microbial production. Microbial systems offer a renewable, sustainable and scalable platform for producing specific polyunsaturated fatty acids (PUFAs) under controlled conditions, providing an eco-friendly alternative to traditional sources. These microorganisms can generate lipids enriched with PUFAs of high nutritional and pharmaceutical relevance, such as γ-linolenic acid (GLA), dihomo-γ-linolenic acid, arachidonic acid (ARA), docosahexaenoic acid (DHA) and eicosapentaenoic acid (EPA), compounds essential for human health and widely used in functional foods and medical formulations (*8*, *16*). The profiles of SCOs can vary significantly, reflecting both strain-specific characteristics and substrate types (*17*). Certain microbial strains have been shown to produce significant amounts of valuable PUFAs, such as DHA and EPA, which are essential for human nutrition (*18*, *19*). For example, the marine oleaginous thraustochytrid *Aurantiochytrium* sp. T66 (ATCC PRA-276) has been reported to efficiently produce polyunsaturated fatty acids, particularly DHA, from volatile fatty acids, reaching up to 42.6 % of total lipids and demonstrating the potential of non-yeast oleaginous microorganisms for sustainable PUFA production (*20*).

Substrate selection is crucial for both lipid yield and fatty acid composition. Using diverse carbon sources from simple sugars to agricultural byproducts and lignocellulosic biomass can significantly affect the balance of saturated, monounsaturated and polyunsaturated fatty acids produced. Simple sugars provide predictable growth and lipid profiles, while food waste valorization offers a sustainable, low-cost alternative. This highlights the versatility of oleaginous microbes for industrial SCO production. Strains such as *Yarrowia lipolytica*, known for their ability to utilize a wide variety of substrates, are promising options for large-scale production of lipids enriched in specific fatty acids with important industrial and nutritional applications. This microorganism has been metabolically engineered to achieve high SCO yields and improved product specificity. The ω‐3 fatty acids produced by *Yarrowia lipolytica* mainly consist of DHA, EPA and α-linolenic acid (ALA) (*21*).

Table 1 (*22*) shows microbial PUFAs, their producers and related companies that have successfully commercialized microbial oils rich in polyunsaturated fatty acids (PUFAs). Martek Biosciences, later acquired by DSM, pioneered large-scale production of DHA and ARA for infant nutrition using *Crypthecodinium cohnii* and *Mortierella alpina*. DuPont engineered *Yarrowia lipolytica* to produce EPA-rich oils, achieving Generally Recognized As Safe (GRAS) status for food applications. These microbial platforms have proven economically viable in the nutraceutical market, especially for high-value lipid products, athough their use as biodiesel feedstocks remains limited due to high production costs and scalability challenges (*23*). Table 1 summarises the most commercially relevant microbial producers of long-chain PUFAs, including ω-3 (DHA and EPA) and ω-6 (ARA and GLA) families (*22*). These compounds are primarily derived from oleaginous microalgae and filamentous fungi cultivated by leading biotechnology companies such as DSM Firmenich, Corbion, Veramaris, and CABIO.

**Table 1:** Most applicable types of microbial polyunsaturated fatty acids (PUFAs) and their producer companies (*22*)

Company	Country	Microorganism	Fatty acid	Brand name (if any)	Main application
DSM	Netherlands	*Mortierella alpina*	ARA	ARASCO™	Infant formula, dietary supplements
Martek Biosciences (part of DSM)	USA	*Crypthecodinium cohnii*	DHA	DHASCO™	Infant formula, food supplements
Martek Biosciences	USA	*Schizochytrium* sp.	DHA	DHASCO™ (also for general food/feed)	Adult nutrition, animal feed
Suntory	Japan	*Mortierella alpina*	ARA	Not specified in reference	Infant nutrition, health supplements
Wuhan Alking Bioengineering	PR China	*Mortierella alpina*	ARA	Not specified in reference	Infant formula, supplements
Lion Corporation (historically)	Japan	*Mortierella alpina*	ARA	Not specified (early developer)	Cosmetics, food (initially for chicken flavour)
Various/historical	-	*Mucor circinelloides*	GLA	Oil of Javanicus (discontinued)	Formerly as GLA source (replaced by borage oil)

DSM is the world's leading producer of ARASCO™, under licence to Martek. ARA (arachidonic acid) and DHA (docosahexaenoic acid) are commercially the most successful microbial single-cell oils (SCOs), primarily used in infant formula. DHASCO™ is produced from the microalgae *Crypthecodinium cohnii* and approved for infant nutrition in many countries. GLA (γ-linolenic acid) production from *Mucor circinelloides* (oil of Javanicus) was discontinued due to competition with plant-based sources like borage oil. Many infant formula manufacturers (*e.g.* Abbott, Nestle, Mead Johnson) use ARASCO™ and DHASCO™ under licence

## MICROBIAL PRODUCTION OF POLYHYDROXYALKANOATES

Polyhydroxyalkanoates (PHAs) are a class of biodegradable polymers synthesized by various bacteria and some archaea under limited nutritional conditions as intracellular carbon and energy storage compounds (*22*, *24*). These biopolymers have attracted considerable interest due to their biodegradability, biocompatibility and potential to replace petrochemical plastics in diverse applications (*24*). The synthesis of PHAs is highly dependent on the availability and type of carbon source; lipid-based substrates and fatty acids can serve as precursors for PHA monomer synthesis, linking lipid metabolism and polymer production (*6*, *25*). Key enzymes, including β-ketothiolase, acetoacetyl-CoA reductase and PHA synthase, can be modulated to enhance PHA accumulation under food-waste-derived fermentation conditions (*7*). Liu *et al.* (*7*) comprehensively reviewed microbial strategies for converting food-derived substrates, such as volatile fatty acids (VFAs) and hydrolysates, into PHAs, emphasizing the influence of nutrient limitation and carbon source optimization on polymer yield (*7*).

## PHA *VS* SCO: DUAL POTENTIAL OF OLEAGINOUS MICROORGANISMS

Both SCOs and PHAs are intracellular carbon storage compounds, mostly produced by oleaginous eukaryotes (yeasts and filamentous fungi) and prokaryotes, respectively, but they differ significantly in chemistry and physical properties. SCOs predominantly consist of triacylglycerols (TAGs), which mimic plant or animal lipids in structure and functionality, having a glycerol backbone esterified with three long-chain fatty acids (C_14_ to C_24_). This structure makes them suitable for a range of applications, including biodiesel production, cosmetics and functional foods (*17*, *18*). These fatty acids can be saturated or unsaturated (*e.g*. oleic, linoleic, DHA), with unsaturation reducing intermolecular packing and keeping SCOs liquid at ambient temperature (*16*, *23*). In contrast, PHAs are biodegradable polyesters, stored as granules in the microbial cytoplasm formed by enzymatic polymerization of (R)-3-hydroxy fatty acid monomers, such as 3-hydroxybutyrate or 3-hydroxyvalerate (*6*, *24*, *26*). Unlike TAGs in SCOs, which serve as short-term energy reserves in oleaginous yeasts, fungi and algae, PHAs are high-molecular-mass polymers synthesized mainly by bacteria under nutrient-limited conditions, serving as long-term carbon and energy storage (*12*).

Structurally, TAGs are hydrophobic lipids suited for nutrition and biofuel applications, while PHAs, due to their polyester nature, are biodegradable and biocompatible, making them ideal for bioplastics and medical uses. The fatty acid composition in SCOs influences properties like oxidative stability and melting point, while the monomer composition in PHAs affects polymer flexibility, crystallinity and mechanical strength (*16*, *23*).

Metabolically, SCO accumulation occurs *via de novo* fatty acid synthesis, regulated by enzymes such as adenosine triphosphate citrate lyase (ACL) and malic enzyme (ME), especially under nitrogen limitation, which redirects carbon flux from biomass to lipid synthesis (*23*). Similarly, PHA synthesis is governed by PHA synthase enzymes using substrates like acetyl-CoA or propionyl-CoA under nutrient-limited conditions (*6*, *12*, *24*). Similar metabolic shifts under nitrogen limitation also trigger PHA accumulation in food-waste-fed bacteria, suggesting overlapping regulatory signals for both lipid and polymer biosynthesis (*7*). Several oleaginous microorganisms (classified as the border zone of producers) have both metabolic pathways of PHA and SCO production and are capable of producing both SCOs and PHAs, making them promising candidates for integrated bioprocesses (*27*). These microorganisms, which belong to actinobacteria, include *Rhodococcus* spp., *Streptomyces* spp. or some species of *Bacillus,* such as *Bacillus subtilis* (*11*, *28*-*32*).

## STRATEGIES FOR LOW-COST INTEGRATED BIOPROCESS DEVELOPMENT USING FOOD WASTE VALORIZATION

Oleaginous microorganisms can produce SCOs and PHAs from various substrates, including sugars, agricultural byproducts and food waste. Liu *et al.* (*7*) demonstrated that optimized fed-batch and nutrient-controlled strategies using food waste hydrolysates increased PHA productivity while reducing operational costs, confirming the economic feasibility of waste-derived systems (*7*).

A study from the University of Anbar (Iraq) showed that *Bacillus subtilis* isolates can efficiently produce SCOs using local soil and environmental wastes as carbon sources (*32*). Palm fronds were identified as the most effective substrate, yielding oils rich in linoleic (46 %) and palmitoleic (16 %) acids. The results highlight the potential of locally sourced, low-cost bacterial systems for sustainable SCO production and food applications.

In the review by Nguyen *et al.* (*10*), various agro-industrial and food waste streams were investigated as alternative substrates for SCO production by oleaginous yeasts. The reported wastes included molasses, sugarcane bagasse hydrolysate, wheat straw, dried sweet sorghum stalks, corn stover hydrolysate, cassava peel waste, apple pomace, vegetable residues, distillery effluents and waste cooking oil. Compared to synthetic media such as glucose- or xylose-based formulations, these waste-derived substrates substantially reduce raw material costs and enhance the overall sustainability of the process by utilizing renewable organic residues. The choice of carbon source strongly influences lipid accumulation and fatty acid composition in oleaginous yeasts. Strains such as *Cutaneotrichosporon oleaginosus* and *Lipomyces starkeyi* achieved the highest SCO yields (up to 30–33 g/L) when cultivated on glucose- or xylose-based media, while the use of agro-industrial residues and crude glycerol offered a more sustainable and cost-effective alternative, still supporting yields in the range of 10–25 g/L. *Yarrowia lipolytica* and *Rhodosporidium toruloides* performed efficiently on low-cost substrates such as molasses, crude glycerol and lignocellulosic hydrolysates, producing oils enriched in mono- and polyunsaturated fatty acids. Although food waste hydrolysates resulted in lower lipid titres (4–10 g/L), they represent an eco-friendly route for circular bioeconomy valorization, emphasizing that substrate selection not only determines lipid yield but also tailors the biochemical profile of SCOs, enabling targeted production for biofuel, food and nutraceutical applications (*10*). Some recent reports have highlighted the valorization of food-derived wastes for microbial lipid production. Småros *et al.* (*33*) achieved 26.1 % lipid accumulation by *Apiotrichum brassicae* grown on dairy side streams, yielding fatty acids comparable to cocoa butter. Likewise, Vemparala *et al.* (*34*) used canteen food waste hydrolysate as substrate for *Candida neerlandica*, obtaining 0.415 g/L lipids with profiles rich in palmitic, oleic and linoleic acids, confirming the potential of food waste-based media as sustainable alternatives to synthetic substrates (*33*, *34*). Recent findings by Dimitriadis *et al.* (*35*) demonstrated that municipal and food waste can serve as cost-effective feedstocks for oleaginous microorganisms such as *Yarrowia lipolytica* and *Rhodococcus opacus*, enabling high lipid accumulation comparable to sugar-based systems. The study emphasized that integrating microbial lipid production with waste management significantly reduces process costs and supports the circular bioeconomy. Moreover, recovered single-cell oils were proposed as dual-purpose intermediates, suitable both for biodiesel synthesis and as substrates for PHA production, reinforcing the potential of dual-target bioprocessing from waste-derived carbon sources (*35*).

Some researchers are exploring new approaches to reduce the downstream processing costs in food waste valorization. Ma *et al.* (*36*) comprehensively analyzed various food waste streams as substrates for microbial lipid production *via* volatile fatty acid (VFA) pathways. The study included fruit and vegetable residues (such as orange, apple and banana peels), kitchen leftovers, bakery waste, dairy effluents, meat processing residues and waste cooking oil. These VFA-rich wastes enabled oleaginous microorganisms like *Yarrowia lipolytica* and *Cutaneotrichosporon oleaginosus* to accumulate lipids with yields ranging from 0.5 to 1.4 g/L, comparable to those obtained from glucose-based systems. In contrast to sugar-rich wastes, which require enzymatic hydrolysis, VFA-based substrates could be directly assimilated, significantly lowering process costs and leaving almost no solid residue. This highlights the superior environmental performance and circular bioeconomy potential of acidogenic food waste valorization for microbial lipid production (*36*).

### Co-production of PHAs and SCOs

In nature, the production of PHAs and SCOs typically occurs separately: oleaginous yeasts, fungi and microalgae are well known for accumulating triacylglycerols (SCOs), while bacteria such as *Cupriavidus necator* are classic producers of poly-(3-hydroxybutyrate) (a type of PHA). However, several oleaginous and metabolically versatile microorganisms have been reported to produce either SCOs or PHAs, and in some cases both, either naturally or through metabolic engineering. Notable examples include *Yarrowia lipolytica* and *Rhodosporidium toruloides*, which are well-known lipid-accumulating yeasts capable of synthesizing triacylglycerols (TAGs) and have been engineered to express PHA biosynthetic pathways (*37*-*39*). Similarly, members of the genus *Rhodococcus* are also recognized for their dual capacity to accumulate both TAGs and PHAs as intracellular carbon and energy reserves (*40*-*42*). In a study by Kumar *et al.* (*43*), efficient co-production of PHAs and carotenoids was achieved by *Paracoccus* sp. strain LL1 using glycerol as the sole carbon source. Under optimized fermentation conditions, the strain produced up to 9.52 g/L of PHA and 7.14 mg/L of carotenoids, demonstrating an integrated bioprocess that enhances the overall economic feasibility of PHA production.

Understanding the metabolic regulation and genetic determinants governing PHA biosynthesis and lipid accumulation enables targeted strain improvement and process optimization. Such dual-production systems have potential to maximize the economic viability and sustainability of microbial fermentation (*24*, *27*). Balancing the metabolic pathways between lipid accumulation and polymer biosynthesis is challenging and requires precise control of culture conditions and substrate feeding strategies (*6*). To address this, metabolic engineering and synthetic biology are increasingly used to optimize these pathways, enabling tailored production of desired compounds and improved yields (*12*, *23*). Such engineered biocatalysts offer great potential for sustainable and economically viable industrial biotechnology.

Table 2 (*30*, *31*, *44*-*48*) summarizes species capable of sequentially producing both PHAs and SCOs in dual-target bioprocessing for the simultaneous production of both compounds.

**Table 2:** Co-production of polyhydroxyalkanoates (PHA) and single-cell oils (SCO) by one microorganism (actinobacteria or bioengineered ones)

Microbial group	Strain/system	Genetic or cultivation strategy	Key result	Reference
Natural “borderline” actinobacteria	*Rhodococcus ruber* NCIMB 40126 *Rhodococcus ruber* PD 630	Grown on glucose under nitrogen limitation	Sequential accumulation: PHBV in early phase, then TAG; approx. 1:1 ratio at the end	(*30*)
	*Rhodococcus aetherivorans* IAR1	Toluene as C-source, N-limited	Simultaneous PHBV and TAG production before N depletion; TAG continued after carbon was exhausted	(*44*)
	*Rhodococcus jostii* RHA1	Genome contains 3 distinct pha clusters	Genomic evidence of dual lipid storage pathways (PHA and TAG) active under different conditions	(*31*)
Minimally or fully engineered oleaginous yeasts	*Yarrowia lipolytica*	Overexpression of *phaC1* from *P. aeruginosa* in peroxisome	Mcl-PHA up to 5 % dry cell mass; yeast still retained strong oil-producing capability	(*45*)
	Engineered *Yarrowia lipolytica*	Multicopy *phaC*, modified β-oxidation	Simultaneous production of mcl-PHA (25–28 % dry cell mass) and natural TAGs	(*46*)
	*Yarrowia lipolytica* PHB32	Full PHB operon + glucose/acetate co-substrate strategy	12 % PHB with high growth rate; confirmed compatibility of PHB and SCO synthesis from cheap feedstock	(*47*)
Other chassis (less common)	*Saccharomyces cerevisiae* (engineered)	Single-copy *phaC* gene; compared to *Y. lipolytica*	mcl-PHA up to 7 % dry cell mass; oil accumulation lower	(*46*)
	*Escherichia coli*	Fed glycerol+FA	Achieved ~0.5 g/L mcl-PHA (~6 % dry cell mass); partial TAG synthesis observed	(*46*, *48*)

“Borderline” refers to microorganisms with the ability to accumulate both TAG and PHA or switching between the two. PHB=polyhydroxybutyrate, PHBV=polyhydroxybutyrate hydroxyvalerate, TAG=tracylglycerol

Both PHA and TAG biosynthetic pathways rely on common precursor molecules, such as acetyl-CoA, fatty acids, or reducing equivalents like nicotinamide adenine dinucleotide phosphate (NADPH). When both pathways are active, they compete for these limited resources, which can reduce the yield of one product if the other predominates. The biosynthesis of lipids and biopolymers involves redox reactions that require a balanced supply of NADH and NADPH. Imbalances in redox cofactors can disrupt metabolic fluxes, leading to inefficient production or accumulation of undesired intermediates. The carbon to nitrogen ratio in the medium is a key regulator of both TAG and PHA synthesis. Nitrogen limitation often triggers storage polymer accumulation. However, simultaneous production of both compounds requires careful adjustment of the C/N ratio to ensure neither pathway is suppressed while maintaining sufficient cell growth (*6*, *12*, *23*).

An alternative strategy to enhance bioprocess efficiency and reduce overall costs is to use two oleaginous microbial species, each contributing to SCO production within an integrated biocatalytic framework. In such systems, the SCOs produced by one organism can serve as a carbon-rich feedstock for a second strain engineered or selected for PHA biosynthesis (*49*).

Notably, PHAs are a family of biodegradable and biocompatible polyesters with increasing applications in food packaging and biomedical sectors (*50*). The integration of microbial lipid and biopolymer production represents a dual-target bioprocessing strategy, particularly when using low-cost or waste-based substrates, and aligns with the principles of industrial sustainability (*6*). The overall success of this approach depends on factors such as strain selection and compatibility, the nature of the carbon source, and the cultivation mode (*e.g.* batch, fed-batch, or continuous), all of which significantly affect lipid yields and fatty acid composition (*51*).

## ANALYTICAL METHODS TO DISTINGUISH PHA AND SCO

In dual-target bioprocesses where both SCOs and PHAs are produced by oleaginous microorganisms, accurately distinguishing between these two intracellular storage compounds is essential. This distinction is critical not only because of their structural and functional differences, but also due to their distinct downstream processing, extraction methods and industrial applications. While both are typically synthesized under nutrient-limited, carbon-rich conditions, PHAs are high-molecular-mass polyesters and solid at room temperature, whereas SCOs consist mainly of triacylglycerols and remain liquid (*52*).

Several analytical techniques have been developed to differentiate PHAs from SCOs. These include fluorescent staining methods, solubility assays, Fourier-transform infrared (FTIR) spectroscopy, gas chromatography–mass spectrometry (GC-MS), and transmission electron microscopy (TEM) (*43*, *52*-*54*). Among staining techniques, Nile Blue A specifically binds to polyhydroxybutyrate (PHB) and fluoresces pink under UV light, while Nile Red preferentially stains neutral lipids with yellow or orange fluorescence (*42*, *52*, *53*). Solubility tests also provide reliable differentiation: SCOs are soluble in cold acetone and chloroform, while PHB dissolves only in hot chloroform and precipitates in cold methanol.

FTIR spectroscopy offers a rapid and non-destructive tool to chemically distinguish these compounds. PHB typically exhibits a strong ester carbonyl peak around 1720 cm^-1^, while SCOs show additional peaks at ~1740 and ~2920 cm^-1^ corresponding to triglyceride esters and aliphatic chains, respectively. GC-MS analysis further supports compound identification; after acid-catalyzed methanolysis, PHB degrades to crotonic acid methyl ester (*m*/*z*=86), while SCOs yield fatty acid methyl esters (FAMEs), indicative of lipid composition (*43*, *54*).

Combining multiple complementary techniques, such as staining, spectroscopy, solubility, and chromatography, not only ensures accurate compound identification but also enhances process monitoring, particularly in integrated biorefineries aiming for the simultaneous production of lipids and biopolymers from renewable substrates.

## CHALLENGES AND OPPORTUNITIES IN DUAL-TARGET BIOPROCESSING

While the potential for dual-target bioprocessing is significant, several challenges must be addressed to optimize yield and efficiency. Key obstacles include substrate variability, metabolic bottlenecks, and the need for coordinated regulatory mechanisms in co-production pathways. Optimizing growth conditions, such as nutrient ratios and fermentation parameters, is crucial for enhancing product yields. Moreover, integrating novel bioprocess strategies, including co-culturing techniques, genetic engineering, and innovative fermentation technologies, can facilitate improved co-production of SCOs and PHAs. Research into techno-economic analyses can help delineate the feasibility of industrial-scale applications, exploring not only financial but also environmental sustainability (*55*-*58*).

Inconsistent nutrient composition of food waste and limited downstream purification efficiency remain the main challenges for stable and scalable production (*19*). Furthermore, improving product recovery efficiency is crucial for achieving economically viable dual-product bioprocesses, particularly when both lipid and polymeric compounds are targeted. As highlighted by Nguyen *et al.* (*10*), one of the major challenges in single-cell oil production is the limited recovery yield caused by inadequate cell disruption and inefficient downstream processing. To overcome these limitations, a combination of physical and chemical treatments, such as sonication, thermal or alkaline pretreatments, and bead-assisted homogenization, can be integrated to enhance intracellular lipid release. Similarly, process optimization strategies including adaptive evolution, nutrient feeding control and metabolic engineering can increase both biomass productivity and product yield. Such integrated approaches not only improve lipid recovery but can also be adapted for the co-extraction of other intracellular metabolites, thereby maximizing the overall process efficiency in dual-production systems (*10*). Evidence from recent studies confirms that microbial valorization of food waste into PHAs offers a viable route towards resource-efficient circular bioeconomy models (*7*).

## FUTURE DIRECTIONS AND PERSPECTIVE

The convergence of microbial lipid and biopolymer production represents a significant advance in sustainable industrial biotechnology, especially when using low-cost feedstocks like food waste. Dual-target bioprocessing, which enables the simultaneous production of SCOs and PHAs, offers substantial benefits in resource efficiency, cost reduction and environmental sustainability. Future research should focus on optimizing metabolic fluxes in native or engineered oleaginous strains for concurrent synthesis. It is suggested that combining microbial consortia with metabolic rewiring approaches can enhance carbon flux partitioning towards PHA biosynthesis from food waste, paving the way for next-generation integrated bioprocesses (*8*).

Synthetic biology and metabolic engineering will be essential for precisely tuning pathways, balancing cofactors, and dynamically regulating processes to maximize yields without sacrificing growth or stability. Integrating advanced analytical tools with bioreactor automation and *in situ* sensing will improve product monitoring, quality control, and scalability.

Emerging biorefinery concepts emphasize complete biomass valorization through cascade utilization, directing lipids to biofuels or nutraceuticals and using residual biomass for PHA or microbial protein production. Multi-stage or co-cultivation systems with tailored microbial consortia can further enhance substrate use and product diversity. While biotechnological valorization focuses on microbial conversion of food wastes into biopolymers and lipids, green extraction approaches such as microwave- and ultrasound-assisted methods have also been developed to recover bioactive compounds and essential oils from food processing byproducts, expanding the circular bioeconomy (*59*).

Commercial success depends on supportive policies, circular bioeconomy incentives, and life cycle assessments confirming environmental and economic advantages over fossil-based alternatives. Overall, the evolution of dual-target microbial bioprocesses aligns with sustainable development, waste valorization and industrial decarbonization, positioning this field as a cornerstone of future bio-manufacturing through interdisciplinary innovation.

## CONCLUSIONS

The use of oleaginous microorganisms for the dual-target bioprocessing of food waste into single-cell oils (SCOs) and polyhydroxyalkanoates (PHAs) offers a valuable opportunity to mitigate resource depletion and pollution while addressing the growing demand for sustainable bio-based products. Aligning this approach with circular bioeconomy principles enables better utilization of low-cost substrates and reduces the overall environmental footprint of production processes. Continued research is necessary to overcome existing challenges and optimize integrated bioprocessing strategies, thereby supporting a transition to a more sustainable and resource-efficient bioeconomy.
